# Association of CD8^+^TILs co-expressing granzyme A and interferon-γ with colon cancer cells in the tumor microenvironment

**DOI:** 10.1186/s12885-024-12605-y

**Published:** 2024-07-19

**Authors:** Jiayi Yang, Xinyi Ding, Zhang Fang, Shaoxian Wu, Maoling Yuan, Rongzhang Chen, Qinlan Xu, Xinran Gao, Haoyu Wu, Lujun Chen, Xiao Zheng, Jingting Jiang

**Affiliations:** 1 Department of Tumor Biological Treatment, the Third Affiliated Hospital of Soochow University, Jiangsu Changzhou, Jiangsu 213003 China; 2 Jiangsu Engineering Research Center for Tumor Immunotherapy, the Third Affiliated Hospital of Soochow University, Changzhou, Jiangsu 213003 China; 3 Institute of Cell Therapy, the Third Affiliated Hospital of Soochow University, Changzhou, Jiangsu 213003 China; 4 Department of Gastroenterology, the Third Affiliated Hospital of Soochow University, Changzhou, Jiangsu 213003 China

**Keywords:** Granzyme A, GSDMB, Colon cancer, Interferon-γ, CD8^+^T cells

## Abstract

CD8^+^T cells secreting granzyme A (GZMA) can induce pyroptosis in tumor cells by effectively cleaving gasdermin B (GSDMB), which is stimulated by interferon-γ (IFN-γ). However, the interaction between GZMA-expressing CD8^+^T cells and GSDMB-expressing tumor cells in colon cancer remains poorly understood. Our research employed multi-color immunohistochemistry (mIHC) staining and integrated clinical data to explore the spatial distribution and clinical relevance of GZMA- and IFN-γ-expressing CD8^+^ tumor-infiltrating lymphocytes (TILs), as well as GSDMB-expressing CK^+^ cells, within the tumor microenvironment (TME) of human colon cancer samples. Additionally, we utilizing single-cell RNA sequencing (scRNA-seq) data to examine the functional dynamics and interactions among these cell populations. scRNA-seq analysis of colorectal cancer (CRC) tissues revealed that CD8^+^TILs co-expressed GZMA and IFN-γ, but not other cell types. Our mIHC staining results indicated that a significant reduction in the infiltration of GZMA^+^IFN-γ^+^CD8^+^TILs in colon cancer patients (*P* < 0.01). Functional analysis results indicated that GZMA^+^IFN-γ^+^CD8^+^TILs demonstrated enhanced activation and effector functions compared to other CD8^+^TIL subsets. Furthermore, GSDMB-expressing CK^+^ cells exhibited augmented immunogenicity. Correlation analysis highlighted a positive association between GSDMB^+^CK^+^ cells and GZMA^+^IFN-γ^+^CD8^+^TILs (*r* = 0.221, *P* = 0.033). Analysis of cell-cell interactions further showed that these interactions were mediated by IFN-γ and transforming growth factor-β (TGF-β), the co-stimulatory molecule ICOS, and immune checkpoint molecules TIGIT and TIM-3. These findings suggested that GZMA^+^IFN-γ^+^CD8^+^TILs modulating GSDMB-expressing tumor cells, significantly impacted the immune microenvironment and patients’ prognosis in colon cancer. By elucidating these mechanisms, our present study aims to provide novel insights for the advancement of immunotherapeutic strategies in colon cancer.

## Introduction

The incidence of colon cancer has increased steadily in recent years, with a notable shift toward younger populations [1, 2]. The advent of immunotherapy has revolutionized the treatment landscape for colon cancer, particularly through the employment of immune checkpoint inhibitors (ICIs) [3]. Researches have indicated that programmed cell death 1 (PD-1) monoclonal antibodies, pembrolizumab and nivolumab, are effective in treating metastatic colon cancer in patients who exhibit mismatch-repair deficiency (dMMR) and high microsatellite instability (MSI-H) [4, 5]. In contrast, these therapeutic agents are not effective in patients with proficient mismatch-repair (pMMR) and microsatellites that are either stable (MSS) or show low microsatellite instability (MSI-L) [3, 4]. The underlying mechanisms of this immune resistance are believed to include a low tumor mutational burden and inadequate immune cell infiltration [4]. Moreover, challenges such as limited therapeutic applicability, variable patient response, and severe side effects, such as cytokine release syndrome (CRS) persist [3]. This underscores the urgent need for exploration into novel immunotherapeutic approaches to develop innovative treatment strategies for colon cancer.

A high infiltration rate of CD8^+^TILs within the colon cancer TME correlates with improved patient prognosis [6]. CD8^+^TILs play a critical role in surveilling and eliminating colon cancer cells that spread from the primary tumor site, significantly impacting the prevention of metastasis [7]. Additionally, the presence of CD8^+^TILs in the TME is linked to a better response to ICIs therapy [8], highlighting their potential as predictive biomarkers and effective therapeutic targets in colon cancer [6]. Granzymes (GZMs) from CD8^+^T cells or natural killer (NK) cells can enter target cells through pores formed by perforin, subsequently cleaving GSDM proteins into N-terminal and C-terminal domains [9, 10]. The N-terminal domain forms channels in the cell membrane, causing cytoplasmic swelling, cell membrane rupture, and the release of inflammatory factors and danger signals, which elicit a strong antitumor immune response [11]. Furthermore, GZMs indirectly promote the cleavage of GSDM proteins by activating caspases, which in turn activate GZMs, creating a positive feedback loop that enhances the overall effect [11].

Researches have indicated that GZMA can specifically cleave GSDMB at the Lys244 or Lys229 residues in tumor epithelial cells, thereby inducing pyroptosis [12, 13]. This mechanism may present a promising approach for tumor immunotherapy [14, 15]. Concurrently, IFN-γ, also originating from CD8^+^TILs, upregulates the transcription of GSDMB mRNA, thereby enhancing this cell pyroptosis pathway [12]. IFN-γ enhances the motility of antigen-specific CD8^+^T cells towards their target antigens and downregulates the expression of vascular endothelial growth factor A (VEGFA), leading to decreased blood flow within tumor tissues [16]. In the CT26 model treated with PD-1, the K229A/K244A double mutants at the GSDMB loci resulted in the most pronounced increases in both tumor volume and weight [12]. Conversely, GSDMB has been identified as playing a pivotal role in the metastasis of colon cancer cells to distant organs by modulating cell adhesion and migration processes [17, 18]. Additionally, GSDMB contributes to the development of resistance against chemotherapy and targeted therapies by promoting protective autophagy [19]. In summary, it is crucial to further explore how CD8^+^TILs co-expressing GZMA and IFN-γ regulate GSDMB in colon cancer epithelial cells and their impact on patient prognosis. Moreover, investigating the effects of GZMA^+^IFN-γ^+^CD8^+^TILs and GSDMB^+^ tumor epithelial cells on signaling pathways within the colon cancer TME is essential. Understanding these mechanisms could enable strategic manipulation of their infiltration ratios to enhance antitumor responses.

In this study, we utilized mIHC to examine the spatial distribution and clinical significance of GZMA, IFN-γ, and GSDMB within TME of human colon cancer patients. Leveraging scRNA-seq data from normal colorectal and CRC tissues, we explored the effector functions and interactions among these cellular groups. Our results reveal that CD8^+^TIL co-expressing GZMA and IFN-γ are crucial in shaping the immune microenvironment, particularly affecting tumor cells that express GSDMB. By elucidating these mechanisms, our study offers new insights that could enhance immunotherapeutic strategies in the management of colon cancer.

## Materials and methods

### Patients and tissue specimens

The human colon cancer tissue microarray (TMA, catalog: HColA180Su21) was provided by Outdo Biotech Co., Ltd., Shanghai, China. A total of 94 patients (46 males and 48 females, aged 29 to 86 years) who underwent surgery from July 2012 to April 2014 were enrolled in this study. This study, which involved human participants, was reviewed and received approval from the Clinical Research Ethics Committee at Outdo Biotech (Shanghai, China, SHYJS-CP-1,910,009).

### Multi-color immunohistochemical staining

The mIHC analysis was performed utilizing the PANO 7-plex IHC kit (catalog No. 0004100100, Panovue, Beijing, China) based on the manufacturer’s instructions to identify the different cell subsets expressing GZMA, IFN-γ, GSDMB, CD8, cytokeratin (CK) on colon cancer and normal colon TMA. CK served to pinpoint malignant epithelial cells, while 4’,6-diamidino-2-phenylindole (DAPI, SIGMA-ALDRICH) highlighted the nucleus. Tissue chips were deparaffinized with xylene, rehydrated with graded ethanol, rinsed in distilled water, and soaked in 10% buffered formalin. Antigen retrieval entailed microwave heating of slides in a citric acid solution, initially at high power, followed by low power for 15 min, with subsequent cooling to ambient temperature. Post-drying, a blocking solution was applied for 10 min at room temperature, succeeded by the incubation with primary antibodies for 1 h. The primary antibodies used were as follows: anti-GSDMB (1:500 dilution, catalog No. ab235540, Abcam, Cambridge), anti-GZMA (1:500 dilution, catalog No. ab209205, Abcam, Cambridge), anti-CD8 (1:400 dilution, catalog No. CST 70,306, Cell signaling technology, USA), anti-IFN-γ (1:800 dilution, catalog No. ab231036-40, Abcam, Cambridge), and anti-CK (1:800 dilution, catalog No. CST4545T, Cell signaling technology, USA). This step was followed by the application of horseradish peroxidase (HRP)-conjugated secondary antibodies for 10 min. Subsequently, the slides were stained with PPD-520 dye (1:100 dilution) for 10 min, washed with TBST, and, if required for additional markers, subjected once more to microwave-mediated antigen retrieval. The final step involved DAPI staining, incubation at room temperature, TBST wash, and mounting with an antifade mounting medium.

### Image analysis

For multispectral imaging, stained slides were scanned using the PanoVIEW VS200 slide scanner (Panovue, Beijing, China) with Olympus 20X lens to create composite stack images. Data was imported into OlyVIA software for spectral unmixing of the multispectral scans, isolating single-channel fluorescence signals. The DAPI staining functioned as an essential navigational tool, facilitating the accurate distinction among the nucleus, cytoplasm, and cell membrane of each cell. The expressions of biomarkers such as CK, CD8, GZMA, GSDMB, and IFN-γ were synthesized with the DAPI staining to produce binary masks. These masks were specifically designed to illuminate cells expressing the aforementioned biomarkers. In particular, the CK binary mask was scrutinized to count local tumor cells effectively. For the purpose of tissue identification, Pan-CK was utilized to differentiate stromal nuclei from epithelial tumor tissue areas, thereby enhancing the precision of tissue recognition.

### Survival analysis and univariate Cox’s model analysis

The fluorescence intensity of each protein across different patients was cut off by surv_cutpoint function of R package survminer, and patients were divided into two groups according to cut off value. Then, Kaplan-Meier survival analysis and univariate Cox’s model analysis were performed using GraphPad Prism 9.5.0 software.

### Single-cell RNA sequencing data analysis

scRNA-seq data from primary, treatment-naïve colorectal cancer (GSE178341) [20] were obtained from the Gene Expression Omnibus (GEO) database. The gene expression profiles and sample metadata were integrated using the Seurat package to construct a Seurat object. Log-normalized data was achieved with the NormalizeData function, and highly variable genes were identified through the FindVariableFeatures function. Data standardization was conducted using the ScaleData function, preparing it for principal component analysis (PCA) with the RunPCA function. Subsequently, uniform manifold approximation and projection (UMAP) was applied for non-linear dimensionality reduction and clustering, emphasizing the leading 20 principal components (PCs) through the RunUMAP function. The clustering analysis on these PCs was conducted using FindNeighbors and FindClusters functions, leveraging the shared nearest neighbor (SNN) algorithm. Visualization of the results was generated using the graphical tools integrated within Seurat.

### Differentially expressed genes and gene sets enrichment analysis

Gene sets were downloaded from the MSigDB database, a comprehensive collection of annotated gene sets available at the website (https://www.gsea-msigdb.org/gsea/*msigdb/*). Subsequently, based on the expression levels of *GZMA* and *IFNG* on CD8^+^TILs, these cells were further stratified into four subgroups: GZMA^−^IFN-γ^−^CD8^+^TILs, GZMA^+^IFN-γ^−^CD8^+^TILs, GZMA^−^IFN-γ^+^CD8^+^TILs, and GZMA^+^IFN-γ^+^CD8^+^TILs. To assess the functional activities of these subgroups, the AddModuleScore function was employed to calculate gene set scores for the Hallmark, Reactome, GOBP, and KEGG datasets. The top 20 pathways showing differential activities were then selected and visualized using the DoHeatmap function to identify key functional differences.

Additionally, epithelial cells were categorized based on the expression of GSDMB into GSDMB^−^ and GSDMB^+^ populations. The FindMarkers function was used to identify differentially expressed genes (DEGs) between these two epithelial cell populations. The clusterProfiler package was then utilized to perform Gene Set Enrichment Analysis (GSEA) on these DEGs, encompassing both KEGG and GO enrichment analyses. The top 15 pathways showing enriched pathways were then selected and visualized using the dotplot function.

### Cell-cell interactions analysis

Gene expression matrices, derived from quality-controlled and standardized single-cell RNA sequencing data, were extracted for CellPhoneDB analysis. The preprocessed data were then subjected to intercellular interaction analysis through the cpdb_statistical_analysis_method function’s statistical analysis module. Then, the ktplots package was utilized for the visualization of the results. By integrating the interaction scores and statistical metrics generated by CellPhoneDB into ktplots, heatmaps and interactive network diagrams were created for visualizations.

### Spatial transcriptome sequencing data analysis

Spatial transcriptome (ST) data from CRC patients [21] were obtained from the Zenodo website (https://zenodo.org/records/7760264) We employed the Load10X_Spatial function to generate Seurat objects from the data. The data were then normalized by the SCTransform function with Log1p normalization and identification of highly variable genes through a Poisson regression model. To address potential batch effects, we integrated multiple datasets using the IntegrateData function. The integrated datasets were then processed using the standard scRNA-seq analysis protocol. We conducted Spearman correlation analysis to investigate the associations among *EPCAM*, *GSDMB*, *CD8A*, *GZMA*, and *IFNG*. For visualizations, we utilized the SpatialFeaturePlot function and the pheatmap package.

### Statistical analysis

Statistical analyses were performed using GraphPad Prism 9.5.0 software. Log-rank survival analysis was used to predict the overall survival (OS) of the patients. Mann-Whitney test was used to analyze the proportion of cells expressing GZMA, GSDMB, IFN-γ, CK and GSDMB in colon cancer and normal colon tissues. Spearman correlation analysis was used to determine the correlation between different cells in the colon cancer microenvironment. *P* < 0.05 was considered statistically significant. Specific details about the significance tests can be found in the accompanying figure legends.

## Results

### GSDMB^+^ epithelial cells regulated by GZMA^+^IFN-γ^+^CD8^+^TILs were enhanced in the TME of CRC

**Fig. 1:**
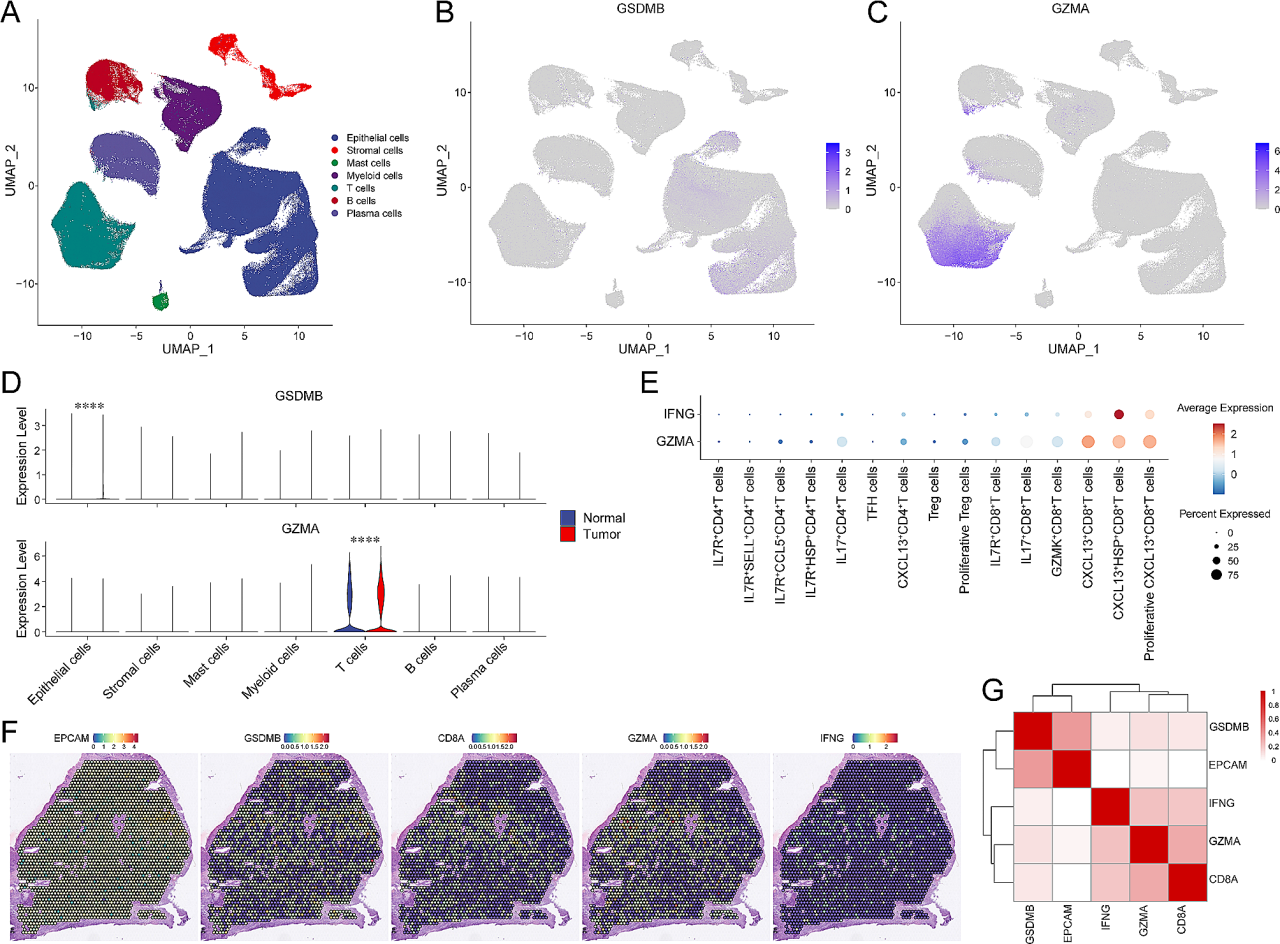
GSDMB^+^ epithelial cells regulated by GZMA^+^IFN-γ^+^CD8^+^TILs were enhanced in TME of CRC. (**A**) UMAP displaying the seven major clusters in normal and CRC tissues: epithelial cells, stromal cells, mast cells, myeloid cells, T cells, B cells, and plasma cells. (**B**-**C**) UMAP showing the expression of *GSDMB* (in **B**) and *GZMA* (in **C**). (**D**) Violin plots compared the expressions of *GSDMB* and *GZMA* across the seven major clusters between normal and CRC tissues. (**E**) Dot plot displaying the expressions of *GZMA* and *IFNG* on CD8^+^TILs. (**F**) SpatialFeaturePlots showing the expressions of *EPCAM*, *GSDMB*, *CD8A*, *GZMA*, and *IFNG.*. (**G**) Heat map displaying Spearman correlation coefficients across the expressions of *EPCAM*, *GSDMB*, *CD8A*, *GZMA*, and *IFNG.*

To clarify and refine the expression of GSDMB, GZMA, and IFN-γ in CRC and normal colorectal tissues, we analyzed scRNA-seq data of both CRC and normal colorectal samples obtained from the GEO database. According to the official standard protocol, 371,223 cells were mainly categorized into seven clusters: epithelial cells, stromal cells, mast cells, myeloid cells, T cells, B cells, and plasma cells (Fig. 1A). We observed that *GSDMB* was mainly expressed on epithelial cells (Fig. 1B), whereas *GZMA* was predominantly expressed on T cells (Fig. 1C). This differential expression pattern implied that T cells might play a significant role in modulating function of GSDMB^+^ epithelial cells by secreting GZMA. Comparative analysis between CRC and normal colorectal tissues revealed a marked increase in *GZMA* within TILs, along with a similar upregulation of *GSDMB* in tumor cells (Fig. 1D). This pattern suggests an enhanced regulatory interaction between GZMA^+^TILs and GSDMB^+^ epithelial cells within the TME. Considering previous findings that IFN-γ, primarily secreted by effector T cells, modulates GSDMB expression in epithelial cells, we evaluated the expression levels of GZMA and IFN-γ among various T cell subsets [22]. Our findings showed that *GZMA* and *IFNG* were notably co-expressed on CXCL13^+^CD8^+^T cells (Fig. 1E). In addition, we utilized spatial transcriptomic data to explore their spatial distribution (Fig. 1F and G). Our findings indicate that *GZMA* and *IFNG* were co-localized within CD8^+^ spots, while *GSDMB* was found within EPCAM^+^ spots. Moreover, we observed co-localization of GZMA and IFNG with GSDMB, suggesting that GZMA^+^IFN-γ^+^CD8^+^T cells might co-localize with GSDMB^+^ epithelial cells. These data demonstrated that GSDMB^+^ epithelial cells, regulated by GZMA^+^IFN-γ^+^CD8^+^T cells, exhibit enhanced activity in the TME of CRC.

### Expression and localization of GSDMB, GZMA and IFN-γ in colon cancer and normal colon tissues

**Fig. 2:**
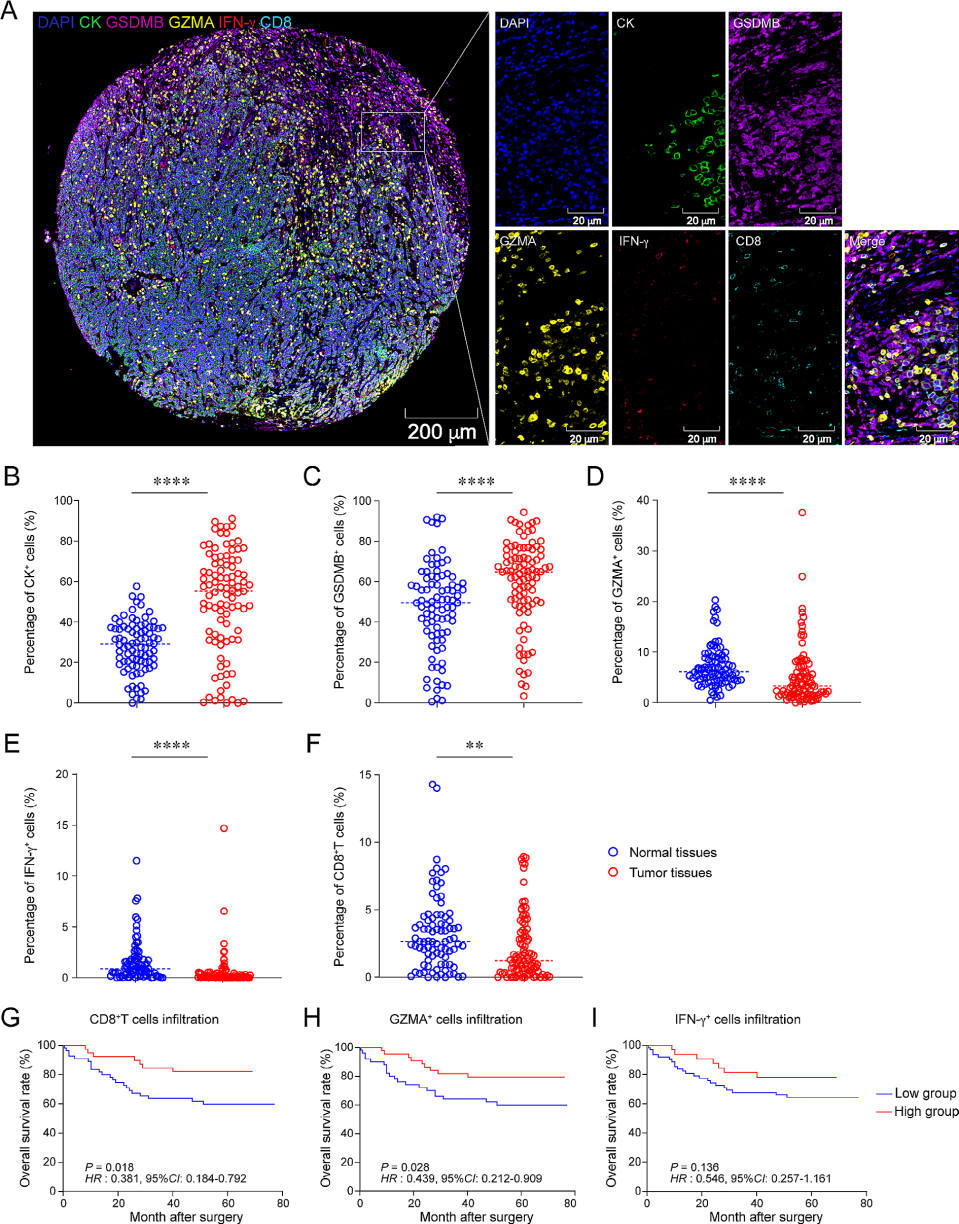
Expression and localization of GSDMB, GZMA and IFN-γ in colon cancer and normal colon tissues. (**A**) mIHC and single-color images were stained in the colon cancer TMA. CK: green, CD8: indigo, GZMA: yellow, GSDMB: purple, IFN-γ: red. (**B**-**F**) Bar plots compared the proportions of cells between normal and tumor tissues. GZMA^+^ cells in (**B**), IFN-γ^+^ cells in (**C**), GSDMB^+^ cells in (**D**), CK^+^ cells in (**E**) and CD8^+^T cells in (**F**). (**G–I**) Kaplan-Meier test analyzed the proportions of CD8^+^T cells (**G**), GZMA^+^ cells (**H**), IFN-γ^+^ cells (**I**) in colon cancer patients. The *P* values in (**G–I**) were calculated by using the Kaplan-Meier test, whereas *HR* and 95%*CI* using univariate Cox’s regression. Statistical analyses in (**B**-**F**) were performed using an unpaired, two-tailed Student’s *t*-test. ^*^P < 0.05, ^***^P < 0.001, ^****^P < 0.0001

To spatially confirm the co-expression of GZMA and IFN-γ in CD8^+^T cells and the expression of GSDMB in epithelial cells, we performed mIHC staining on both colon cancer and normal colon TMA (Fig. 2A: CK (green), CD8 (indigo), GZMA (yellow), GSDMB (purple) and IFN-γ (red)). mIHC staining results revealed that the elevated frequencies of CK^+^ and GSDMB^+^ cells were detected in colon cancer tissues relative to normal colon tissues (Fig. 2B and C, *P* < 0.001). In contrast, the expression of GZMA and IFN-γ in normal colon tissues were significantly upregulated compared to those in colon cancer tissues (Fig. 2D and E, *P* < 0.001). Additionally, the proportion of CD8^+^TILs was also higher in normal colon tissues than that in colon cancer tissues (Fig. 2F, *P* < 0.01).

Cytotoxic CD8^+^TILs, as the primary cell type responsible for combating tumors, can serve as predictors of clinical outcomes in CRC patients. To confirm the prognostic significance of CD8^+^TILs in colon cancer, we assessed the clinical outcome values associated with CD8^+^TILs, GZMA^+^ cells and IFN-γ^+^ cells. Survival analysis results indicated that a higher proportion of CD8^+^TILs in colon cancer was associated with a better prognosis of the patients (Fig. 2G, *HR* = 0.381, 95% *CI*: 0.184–0.792, *P* = 0.018). Similarly, higher frequency of GZMA^+^TILs was significantly associated with better clinical outcomes (Fig. 2H, *HR* = 0.439, 95% *CI*: 0.212–0.909, *P* = 0.028). However, we found no significant association between the frequency of IFN-γ^+^ cells in colon cancer tissues and OS (Fig. 2I, *HR* = 0.546, 95% *CI*: 0.257–1.161, *P* = 0.136). These data suggest that CD8^+^TILs expressing GZMA could be serve as an independent prognosis predictor in colon cancer patients.

### mIHC staining of CD8^+^TILs expressing GZMA and/or IFN-γ in human colon cancer tissues

**Fig. 3:**
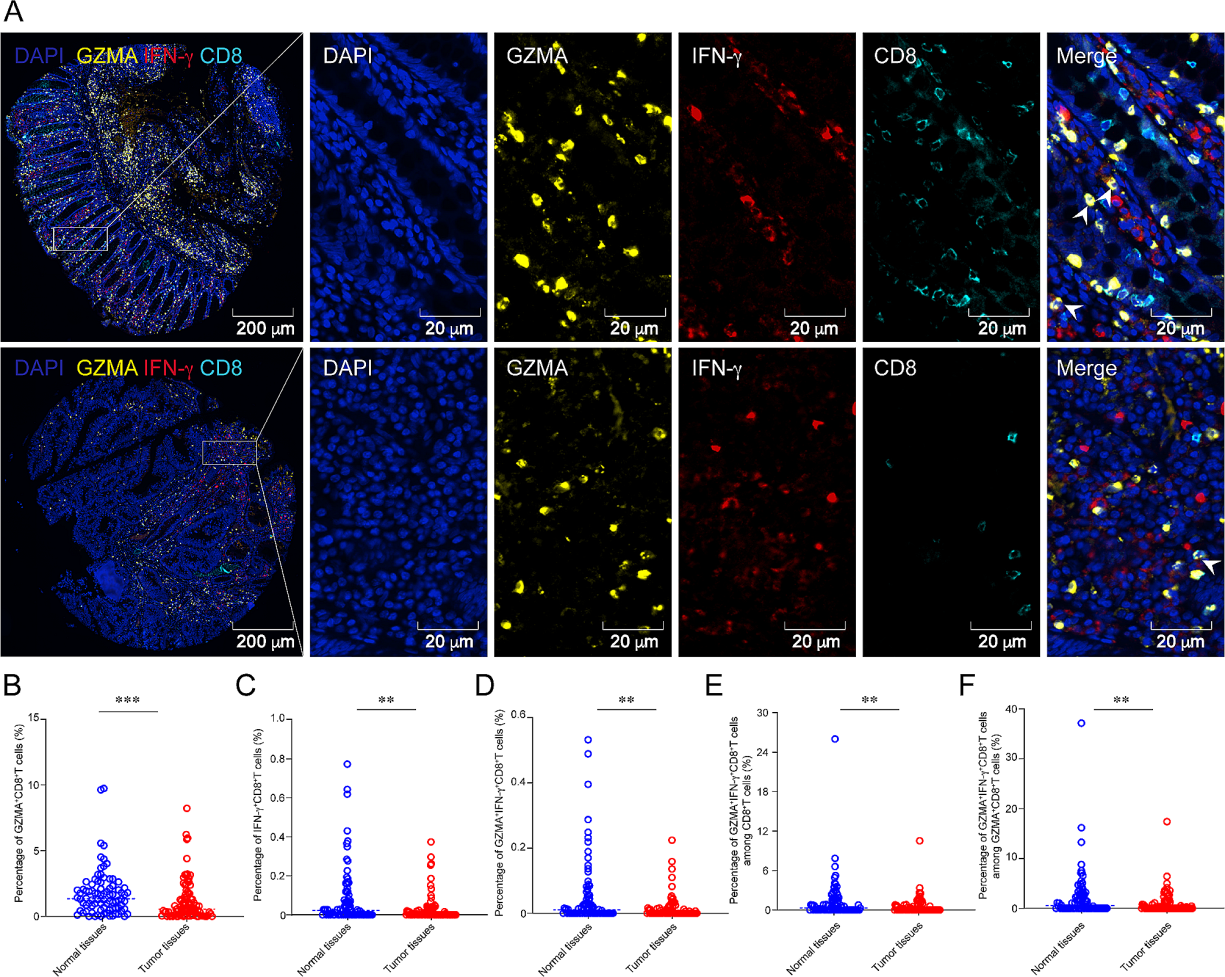
mIHC staining of CD8^+^TILs expressing GZMA and/or IFN-γ in human colon cancer tissues. (**A**) mIHC and single-color images were stained for GZMA^+^IFN-γ^+^CD8^+^T cells in the colon cancer TMA, and the arrows correspond to the GZMA^+^IFN-γ^+^CD8^+^T cells. CD8: indigo, GZMA: yellow, IFN-γ: red. (**B**-**D**) Bar plots compared the proportions of cells between normal and tumor tissues. GZMA^+^CD8^+^T cells in (**B**), IFN-γ^+^CD8^+^T cells in (**C**), GZMA^+^IFN-γ^+^CD8^+^T cells in (**D**). (**E** and **F**) Bar plots compared the proportions of cells between normal and tumor tissues. The ratio of GZMA^+^IFN-γ^+^CD8^+^T cells among CD8^+^T cells in (**E**), and the ratio of GZMA^+^IFN-γ^+^CD8^+^T cells among GZMA^+^CD8^+^T cells in (**F**) between normal colon tissues and colon cancer tissues. Statistical analyses in (**B**-**F**) were performed using an unpaired, two-tailed Student’s *t*-test. ^*^P < 0.05, ^***^P < 0.001, ^****^P < 0.0001

Since GZMA^+^IFN-γ^+^CD8^+^T cells may regulate GSDMB expression on epithelial cells in the TME, Fig. 3A specifically focused on the co-expression of GZMA and IFN-γ in CD8^+^TILs within colon cancer TMA sections. Then, the prognostic significance of GZMA^+^IFN-γ^+^CD8^+^TILs in human colon cancer tissues was assessed. In comparison to colon cancer tissues, normal colon tissues exhibited a higher frequency of GZMA^+^CD8^+^TILs (Fig. 3B, *P* < 0.001). Likewise, the proportion of IFN-γ^+^CD8^+^ TILs and GZMA^+^IFN-γ^+^CD8^+^TILs were significantly elevated in normal colon tissues than those in colon cancer tissues (Fig. 3C and D, *P* < 0.01). In addition, the proportions of GZMA^+^IFN-γ^+^CD8^+^TILs among CD8^+^TILs or among GZMA^+^CD8^+^TILs were higher in normal colon tissues than those in colon cancer tissues (Fig. 3E and F, *P* < 0.01).

### The proportion of CD8^+^TILs co-expressing GZMA and IFN-γ in scRNA-seq data

**Fig. 4:**
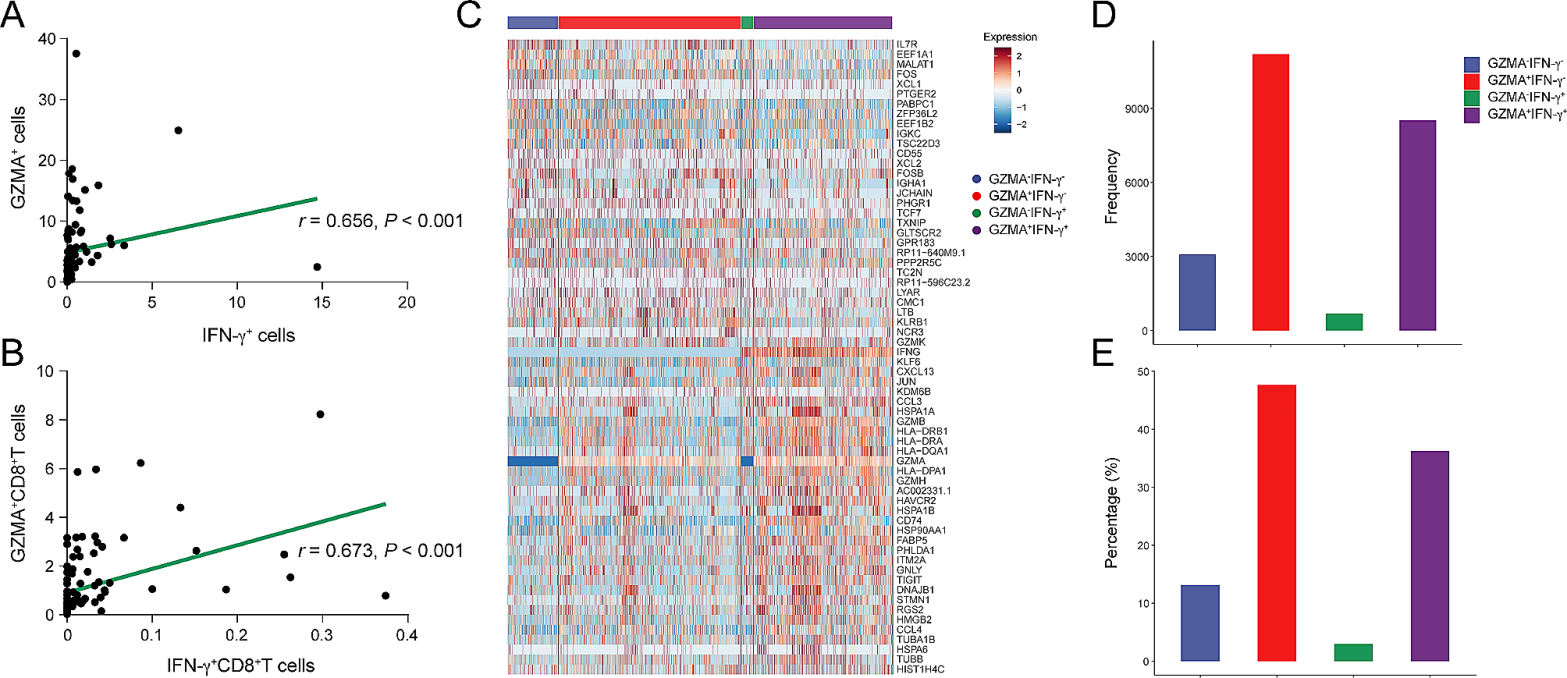
The proportion of CD8^+^TILs co-expressing GZMA and IFN-γ in scRNA-seq data. (**A**) The Spearman correlation for GZMA^+^ cells and IFN-γ^+^ cells. (**B**) The Spearman correlation for GZMA^+^CD8^+^T cells and IFN-γ^+^CD8^+^T cells. (**C**) Heat map displaying the marker genes of each cluster in CD8^+^TILs, which were categorized by the expression profiles of IFN-γ and GZMA. (**D**-**E**) Bar plots showing the frequencies (**D**) and percentages (**E**) of CD8^+^TIL subsets in (**C**)

Expression of IFN-γ in CD8^+^TILs showed no significant correlation with OS, but its expression on GZMA^+^CD8^+^TILs was associated with a better prognosis. Consequently, we further explored the relationship between IFN-γ^+^ cells and GZMA^+^ cells, as well as between IFN-γ^+^CD8^+^TILs and GZMA^+^CD8^+^TILs. Spearman correlation analysis revealed a significant positive correlation between IFN-γ^+^ cells and GZMA^+^ cells (Fig. 4A, *r* = 0.656, *P* < 0.001). Similarly, a positive correlation was observed between the proportions of IFN-γ^+^CD8^+^TILs and GZMA^+^CD8^+^TILs (Fig. 4B, *r* = 0.673, *P* < 0.001). Leveraging the expression profiles of IFN-γ and GZMA, we stratified CD8^+^TILs from scRNA-seq data into four categories: GZMA^−^IFN-γ^−^, GZMA^+^IFN-γ^−^, GZMA^−^IFN-γ^+^ and GZMA^+^IFN-γ^+^ (Fig. 4C). Statistical analyses indicated a high frequency and proportion of GZMA^+^IFN-γ^+^CD8^+^TILs within the total CD8^+^TILs population (Fig. 4D and E).

### The function of GZMA^+^IFN-γ^+^CD8^+^TILs in TME

**Fig. 5:**
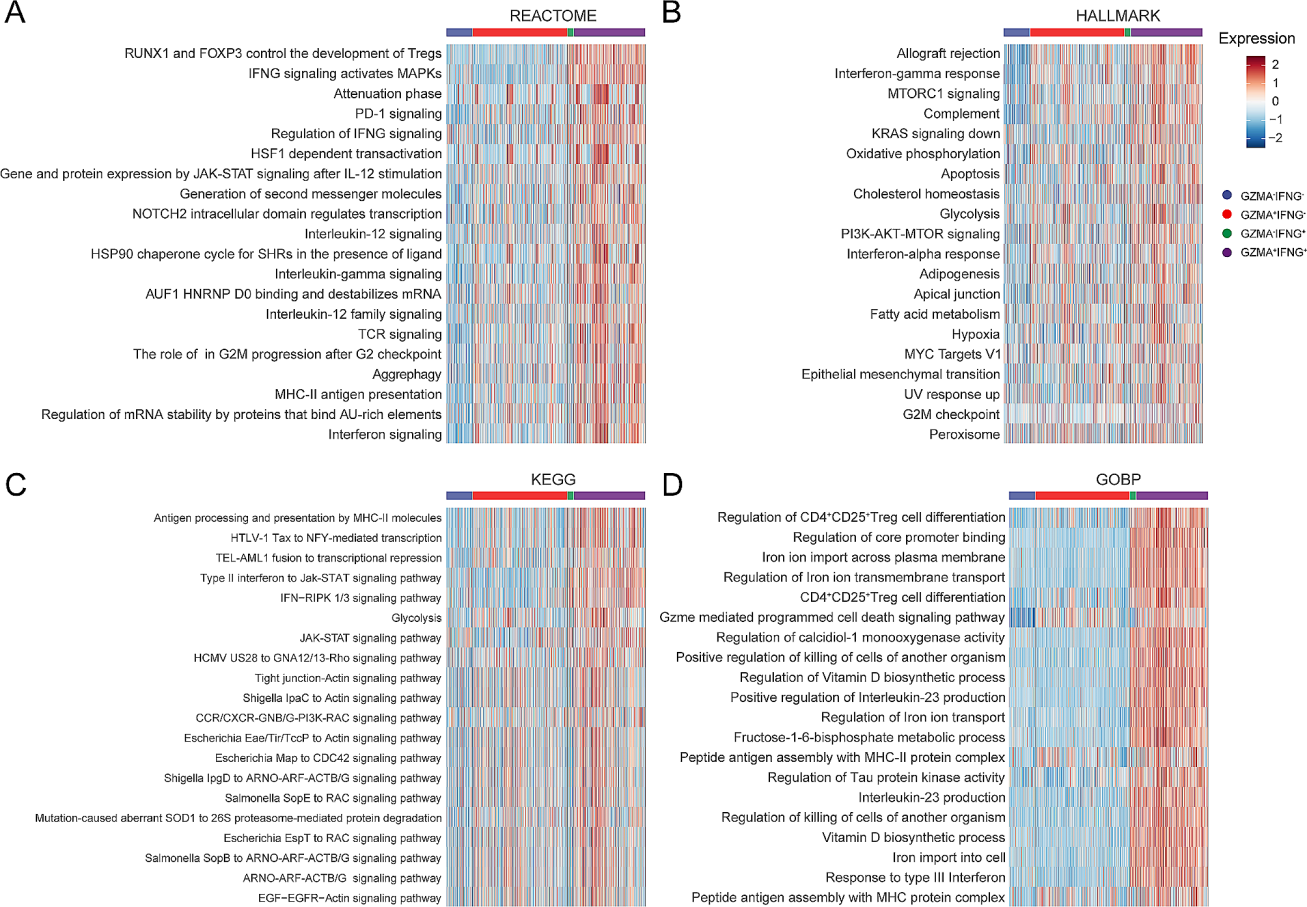
The function of GZMA^+^IFN-γ^+^CD8^+^TILs in TME. (**A**-**D**) Heat maps displaying the top 20 pathways of GSEA results. Reactome (**A**), Hallmark (**B**), KEGG (**C**), and GOBP (**D**)

To further investigate the functional characteristics of GZMA^+^IFN-γ^+^CD8^+^TILs, we conducted GSEA on the total population of CD8^+^TILs in CRC. We sourced gene sets from public databases such as Reactome, Hallmark, KEGG, and GOBP, and computed enrichment scores at the single-cell level. Compared to GZMA^−^IFN-γ^+^CD8^+^TILs, our study identified a pronounced augmentation in the IFN-γ signaling pathway within GZMA^+^IFN-γ^+^CD8^+^TILs, signifying their crucial role in mediating IFN-γ-dependent immune responses (Fig. 5A and D). We also observed upregulation of molecules associated with major histocompatibility complex class II (MHC-II), implying an enhanced capability in antigen presentation and immune surveillance (Fig. 5A, C and D). Furthermore, an increase in the expression of cell cycle-related molecules was detected, possibly reflecting the proliferative dynamics of TILs within the TME (Fig. 5A and C). Metabolic pathways, including glycolysis, oxidative phosphorylation, and fatty acid metabolism, were notably enriched, indicating increased metabolic and energetic adaptations of these cells to the tumor environment (Fig. 5B and C). Through Reactome pathway analysis, we found significant enrichment in IL-12 signaling, TCR signaling, and PD-1 signaling pathways, highlighting their integral role in modulating GZMA^+^IFN-γ^+^CD8^+^TILs functionality (Fig. 5A). Particularly, the enrichment in IL-12 and PD-1 signaling pathways may be pivotal in modulating the immune responsiveness and evasion from the immunosurveillance [23, 24]. In the analysis of the Hallmark pathway, we noted significant enrichment of the PI3K-AKT-mTOR axis in TCR downstream signaling, suggesting active metabolic regulation in signal transduction and reinforcing their dynamic involvement in the TME [25, 26] (Fig. 5B). Altogether, our comprehensive analyses shed light on the intricate functional roles and regulatory mechanisms of GZMA^+^IFN-γ^+^CD8^+^TILs in the tumor microenvironment, underscoring their potential impact on anti-tumor responses.

### Prognostic value of GSDMB^+^CK^+^ cells in human colon cancer

**Fig. 6:**
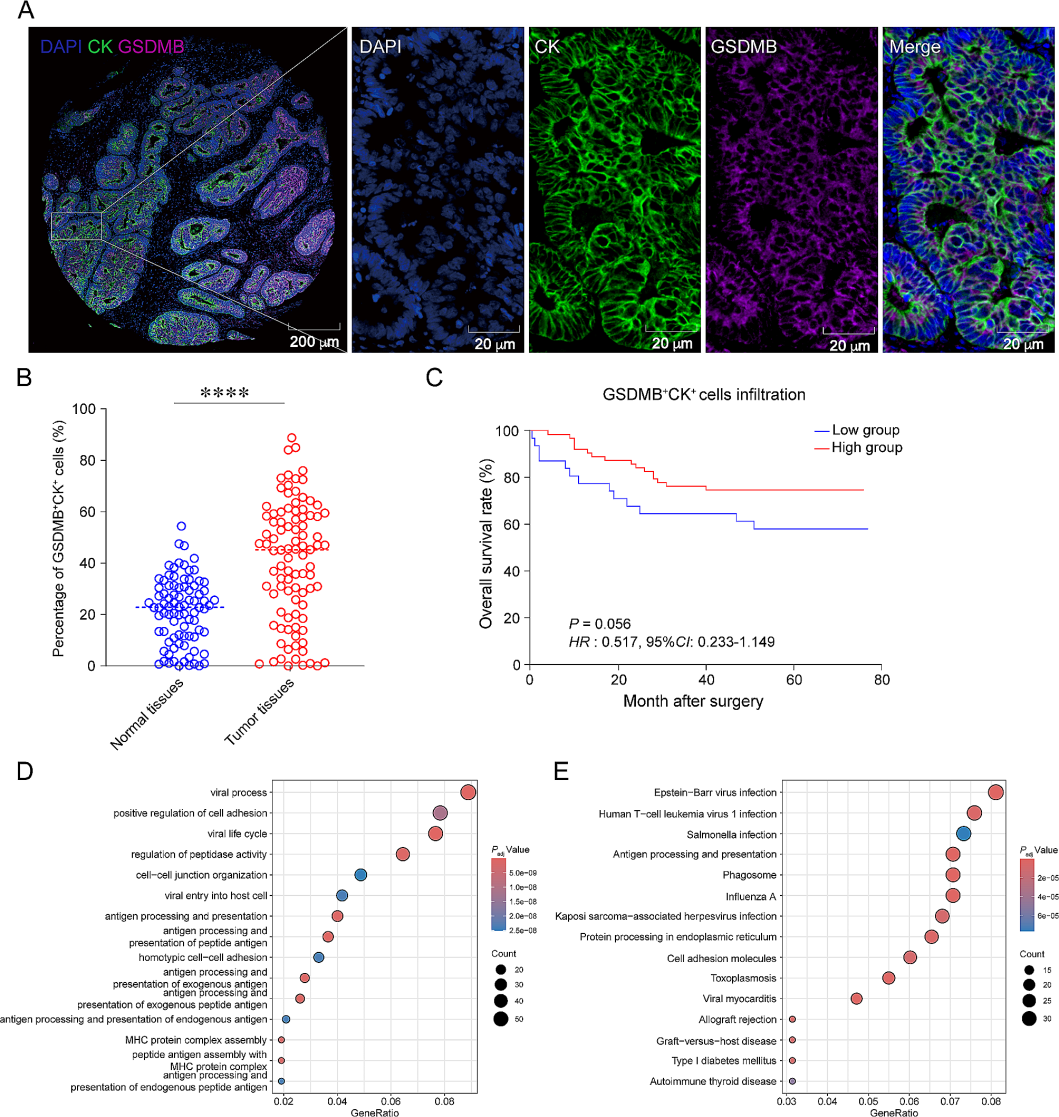
Prognostic value of GSDMB^+^CK^+^cells in human colon cancer. (**A**) mIHC and single-color images were stained in the colon cancer TMA, and the arrows correspond to the GSDMB^+^CK^+^ cells. CK: green, GSDMB: purple. (**B**) Comparison of the proportions of GSDMB^+^CK^+^ cells between normal colon tissues and colon cancer tissues. (**C**) Kaplan-Meier test analyzed the proportions of GSDMB^+^CK^+^ cells in colon cancer patients. (**D** and **E**) Dot plots showing the top 15 pathways of GO and KEGG results. The GO results in (**D**), and KEGG results in (**E**). The *P* value in (**C**) was calculated by using the Kaplan-Meier test, whereas *HR* and 95%*CI* using univariate Cox’s regression. Statistical analysis in (**B**) was performed using an unpaired, two-tailed Student’s *t*-test. ^*^P < 0.05, ^***^P < 0.001, ^****^P < 0.0001

To verify the impact of epithelial cell expressed GSDMB on patients with colon cancer, we calculated the proportion of GSDMB^+^CK^+^ cells and evaluated the prognostic value of GSDMB^+^CK^+^ cells in human colon cancer tissues (Fig. 6A). We found that higher frequency of GSDMB^+^CK^+^ cells was observed in colon cancer tissues compared with normal colon tissues (Fig. 6B, *P* < 0.001). However, we found no significant association between the frequency of GSDMB^+^CK^+^ cells in colon cancer tissues and overall survival (OS) (*HR* = 0.517, 95% *CI*: 0.233–1.149, *P* = 0.056, Fig. 6C).

To elucidate the functional differences between GSDMB^−^CK^+^ and GSDMB^+^CK^+^ cells, we analyzed their differential gene expression profiles and conducted GO and KEGG enrichment analyses (Fig. 6D and E). The results indicated significant enrichment of genes associated with antigen processing and presentation, as well as cell-cell adhesion, in GSDMB^+^CK^+^ cells. These findings suggested that GSDMB^+^CK^+^ cells may play a pivotal role in the pathogenesis of colon cancer, particularly in the immunoregulation of the tumor microenvironment.

### Correlation among IFN-γ^+^CD8^+^TILs, GZMA^+^CD8^+^TILs and GSDMB^+^CK^+^ cells in the TME of colon cancer

**Fig. 7:**
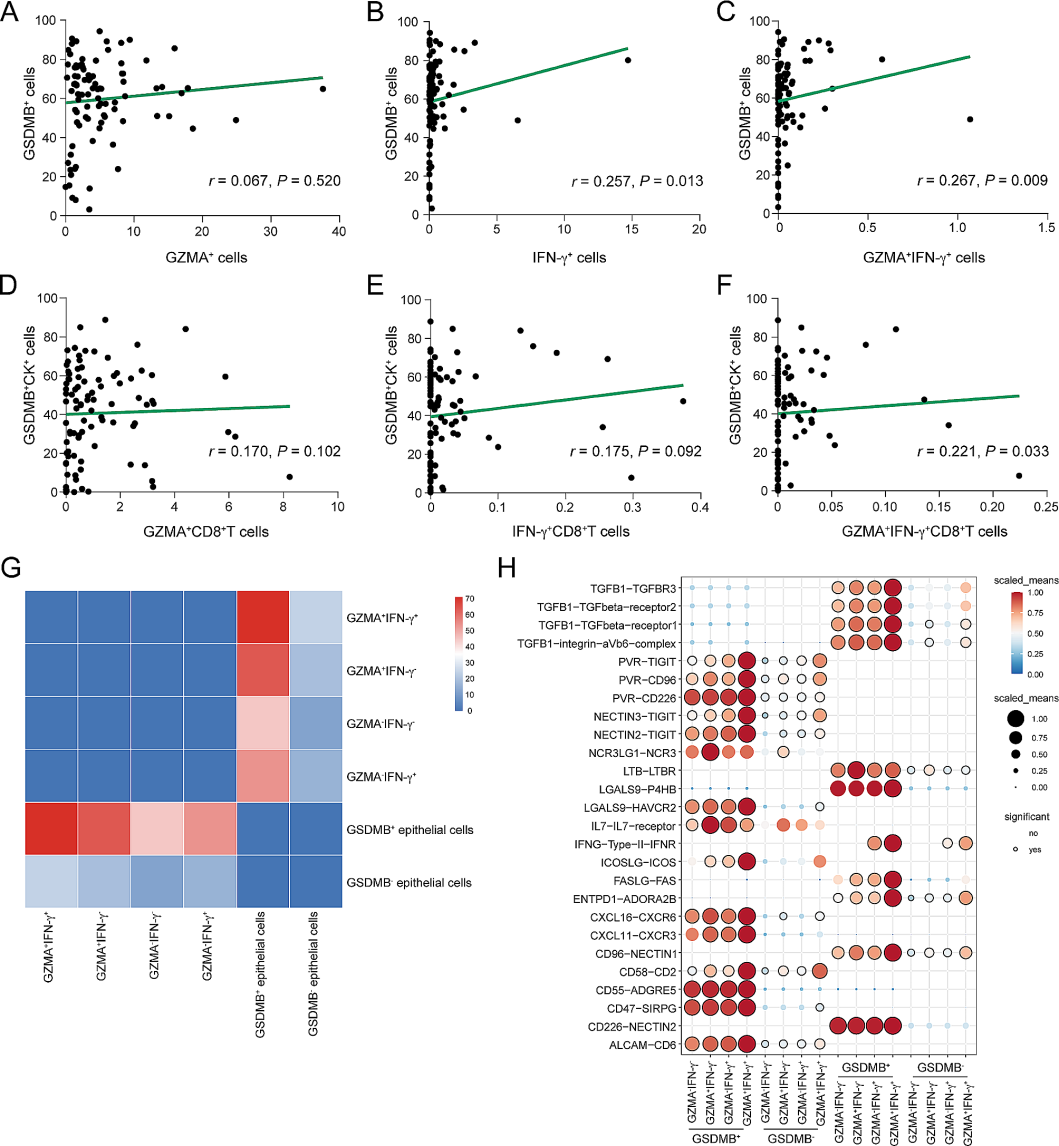
Correlation among IFN-γ^+^CD8^+^TILs, GZMA^+^CD8^+^TILs and GSDMB^+^CK^+^cells in the TME of colon cancer. (**A**-**C**) The Spearman correlations for GZMA^+^ cells and GSDMB^+^ cells in (**A**), IFN-γ^+^ cells and GSDMB^+^ cells in (**B**), GZMA^+^IFN-γ^+^ cells and GSDMB^+^ cells in (**C**). (**D**-**F**) The Spearman correlations for GZMA^+^CD8^+^T cells and GSDMB^+^CK^+^ cells in (**D**), IFN-γ^+^CD8^+^T cells and GSDMB^+^CK^+^ cells in (**E**), GZMA^+^IFN-γ^+^CD8^+^T cells and GSDMB^+^CK^+^ cells in (**F**). (**G**) Heat map displaying the interactions among epithelial subsets and CD8^+^TIL subsets. (**H**) Dot plot showing the strength of ligand-receptor pairs among epithelial subsets and CD8^+^TIL subsets

To further investigate the associations among IFN-γ^+^ cells, GZMA^+^ cells and GSDMB^+^ cells, we conducted correlation analysis. Our results revealed no significant correlation between the infiltration levels of GZMA^+^ cells and that of GSDMB^+^ cells (*r* = 0.067, *P* = 0.520, Fig. 7A). Conversely, a notable positive correlation was observed between the counts of IFN-γ^+^ cells and GSDMB^+^ cells (*r* = 0.257, *P* = 0.013, Fig. 7B), as well as between GZMA^+^IFN-γ^+^ cells and GSDMB^+^ cells (*r* = 0.267, *P* = 0.009, Fig. 7C). Moreover, our analysis showed that neither the proportion of GZMA^+^CD8^+^TILs nor IFN-γ^+^CD8^+^TILs correlated significantly with the ratio of GSDMB^+^CK^+^ cells (*r* = 0.170, *P* = 0.102, Fig. 7D; *r* = 0.175, *P* = 0.092, Fig. 7E). However, a positive correlation was detected between the number of GZMA^+^IFN-γ^+^CD8^+^TILs and GSDMB^+^CK^+^ cells within the colon cancer microenvironment (*r* = 0.221, *P* = 0.033, Fig. 7F). Considering the impact of these factors on colon cancer patients’ survival, it suggested that the signaling pathways involving these markers may play crucial roles in enhancing long-term prognosis for colon cancer patients.

To clarify the intricate interplays between GZMA^+^IFN-γ^+^CD8^+^TILs and GSDMB^+^CK^+^ cells, we utilized the cellphoneDB package, a tool that delineates the interaction networks in cellular communication [27]. Our analysis highlighted a pronounced interaction between GZMA^+^IFN-γ^+^CD8^+^TILs and GSDMB^+^CK^+^ cells, exceeding that in other CD8^+^T cell subsets (Fig. 7G). Notably, GZMA^+^IFN-γ^+^CD8^+^TILs emerged as key regulators of GSDMB^+^CK^+^ cells, with IFN-γ and TGF-β acting as essential mediators in this intercellular dialogue. Additionally, a stronger interaction was observed between FasL produced by GZMA^+^IFN-γ^+^CD8^+^TILs and the Fas receptor on GSDMB^+^CK^+^ cells, enhancing their interconnection. The expression of ICOSL on GSDMB^+^CK^+^ cells also appeared to increase ICOS activation on GZMA^+^IFN-γ^+^CD8^+^TILs, indicating a more vigorous immunologic synergy [28]. Importantly, the immunosuppressive capacity of GSDMB^+^CK^+^ cells on GZMA^+^IFN-γ^+^CD8^+^TILs was amplified, especially through interactions with TIGIT and TIM-3 (*HAVCR2*), underscoring these pathways as pivotal in immune evasion mechanisms [29–31] (Fig. 7H). These detailed insights not only enrich our comprehension of the bidirectional interactions between GZMA^+^IFN-γ^+^CD8^+^TILs and GSDMB^+^CK^+^ cells but also provide a strong theoretical basis for developing targeted immunotherapies against these cell interactions.

## Discussion

**Fig. 8:**
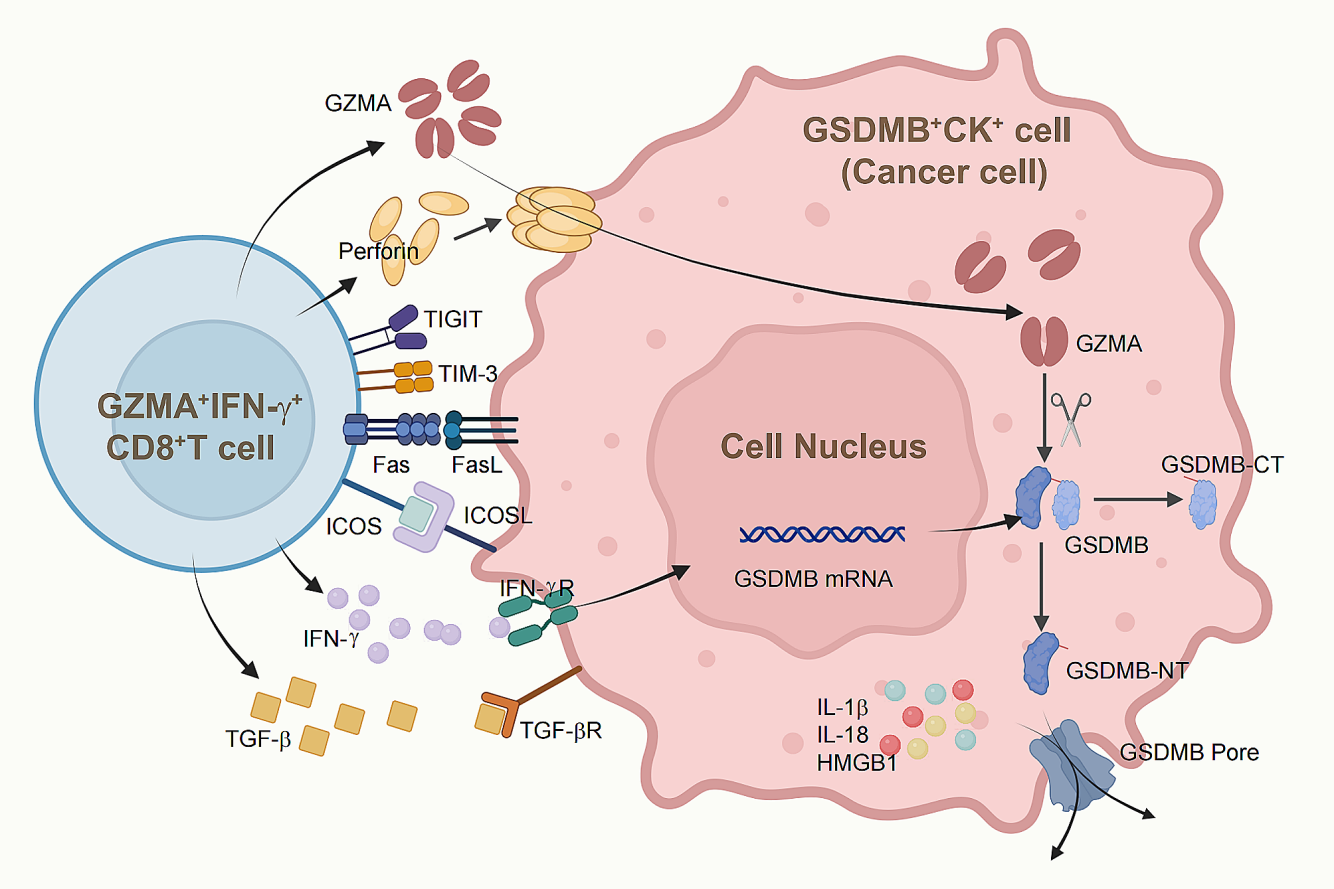
Diagram of the interactions between GZMA^+^IFN-γ^+^CD8^+^TILs and GSDMB^+^CK^+^ cells in the TME of colon cancer. Created with BioRender.com

Research findings demonstrated that GZMA released by CD8^+^TILs can effectively cleave GSDMB within tumor epithelial cells, culminating pyroptosis [12]. Moreover, IFN-γ produced by CD8^+^TILs can bolster GSDMB expression, further intensifying this cascade [12]. Our findings revealed a pronounced expression of GSDMB in colon cancer cells, with GZMA and IFN-γ frequently co-expressed within CD8^+^TILs. Spatial transcriptomics and mIHC analysis revealed that CD8^+^TILs co-expressing GZMA and IFN-γ may localize near GSDMB-expressing tumor cells, suggesting potential interactions between these cell types. Cell-cell interactions analysis results revealed that GZMA^+^IFN-γ^+^CD8^+^TILs form stronger interactions with GSDMB^+^CK^+^ cells compared to other CD8^+^T cell subsets, facilitated by IFN-γ and TGF-β, the co-stimulatory molecule ICOS, and the immune checkpoint molecules TIGIT and TIM-3. These interactions are further enhanced by the engagement of FasL on GZMA^+^IFN-γ^+^CD8^+^TILs with the Fas receptor on GSDMB^+^CK^+^ cells. Notably, the immunosuppressive impact of GSDMB^+^CK^+^ cells on GZMA^+^IFN-γ^+^CD8^+^TILs can be augmented through their interactions with TIGIT and TIM-3 (*HAVCR2*), highlighting the critical roles of these pathways in immune evasion mechanisms [31–33] (Fig. 8).

Notably, GZMA^+^IFN-γ^+^CD8^+^TILs subset demonstrated enhanced activation and effector functions compared to other CD8^+^TIL populations. However, the proportion of GZMA^+^IFN-γ^+^CD8^+^TILs was significantly reduced in colon cancer patients, likely due to immune escape [32]. Additionally, inhibitory cytokines secreted by tumor cells, regulatory T cells (Tregs), or myeloid-derived suppressor cells (MDSCs) within the TME further suppress the proliferation and functionality of GZMA^+^IFN-γ^+^CD8^+^TILs [14]. Persistent tumor antigens in the TME can also lead to T cell exhaustion, characterized by diminished functionality, reduced proliferative capacity, and high inhibitory receptor expression [34].

The IFN-γ signaling pathway was significantly enhanced in GZMA^+^IFN-γ^+^CD8^+^TILs, highlighting their crucial function in mediating IFN-γ-dependent immune responses. Additionally, the elevation of molecules associated with the MHC-II suggested that GZMA^+^IFN-γ^+^CD8^+^TILs enhance antigen presentation, thereby strengthening immune surveillance capabilities. Upon stimulation by antigenic peptide-loaded MHC-II molecules, activated CD4^+^TILs secrete cytokines such as IL-2, which promote the proliferation and differentiation of CD8^+^TILs [35]. Additionally, activated CD8^+^TILs can also express MHC-II molecules, allowing them to present antigens [36]. By regulating the activation, proliferation, and apoptosis of CD8^+^TILs, MHC-II plays a critical role in maintaining immune homeostasis and preventing autoimmune responses [35].

Metabolic pathways such as glycolysis, oxidative phosphorylation, and fatty acid metabolism were notably more active in GZMA^+^IFN-γ^+^CD8^+^TILs. This activity underscores the cells’ heightened adaptability to the metabolic and energetic demands of the TME. The functionality of GZMA^+^IFN-γ^+^CD8^+^TILs was significantly influenced by the IL-12 signaling pathway, TCR signaling pathway, and PD-1 signaling pathway. The IL-12 and PD-1 pathways, in particular, are thought to play key roles in modulating immune responses and evasion from the immunosurveillance [23, 24]. Consequently, we suggest boosting IL-12 expression as a strategy to amplify the infiltration ratio of GZMA^+^IFN-γ^+^CD8^+^TILs, potentially elevating the sensitivity of colon cancer cells to both immunotherapy and chemotherapy, thus intensifying antitumor efficacy. These insights furnish novel perspectives on the mechanisms by which GZMA^+^IFN-γ^+^CD8^+^TILs modulate the TME of colon cancer, providing innovative angles for therapeutic intervention.

Although no statistical correlation was observed between the infiltration ratio of GSDMB^+^CK^+^ cells and patients’ prognosis in colon cancer, these cells exhibit enhanced immunogenic properties, contributing to antigen processing, presentation, and intercellular adhesion. This underscores the multifaceted role of GSDMB^+^CK^+^ cells in the pathogenesis of colon cancer. Correlation analyses identified a positive association with GZMA^+^IFN-γ^+^CD8^+^TILs, suggesting intricate cellular interactions. GSDMB-mediated pyroptosis releases various intracellular inflammatory factors, such as IL-1β and IL-18, thereby enhancing the local inflammatory response and activating immune cells, including GZMA^+^IFN-γ^+^CD8^+^TILs [14]. This activation further stimulates these CD8^+^TILs to secrete increased amounts of GZMA and IFN-γ, thereby enhancing their cytotoxic effects on tumor cells and forming a positive feedback loop [14]. Tumor cells express inhibitory molecules which interact with receptors on these TILs, like TIM-3, and TIGIT, inhibiting their activation and function. Therefore, antagonizing the synergistic effect of TIGIT and TIM-3 offers a potent strategy to enhance the activity and infiltration of GZMA^+^IFN-γ^+^CD8^+^TILs. This approach can significantly boost their anti-tumor immune response, potentially surpassing the efficacy of PD-1 and cytotoxic T lymphocyte-associated antigen-4 (CTLA-4) monoclonal antibody therapies. These findings demonstrate the significant influence of GZMA^+^IFN-γ^+^CD8^+^TILs on the immune microenvironment in colon cancer, which in turn affects patient prognosis by modulating tumor cells expressing GSDMB.

Our research sheds light on the clinical implications and functional roles of CD8^+^T cells expressing GZMA and IFN-γ, as well as colon cancer cells expressing GSDMB. It delved into their intricate mechanisms of interaction, laying the groundwork for novel pathways in prognostic evaluation, therapeutic efficacy monitoring, overcoming resistance to immunotherapy, and crafting immunotherapeutic strategies for colon cancer patients. However, despite selecting an effective IFN-γ antibody for staining, we noted suboptimal immunohistochemical staining of IFN-γ, resulting in an undetectable level of GZMA^+^IFN-γ^+^CD8^+^TILs in many colon cancer samples. This observation introduces a potential statistical bias in assessing the impact of these TILs on patient prognosis. Consequently, we refrained from conducting survival analysis based on this parameter. Future studies should increase the sample size to investigate the correlation between the infiltration of GZMA^+^IFN-γ^+^CD8^+^TILs and the prognosis of colon cancer patients, to determine whether this ratio can serve as a reliable biomarker for prognosis assessment and treatment efficacy monitoring in colon cancer. Moreover, it is imperative for future studies to unravel the precise mechanisms by which GZMA^+^IFN-γ^+^CD8^+^TILs target GSDMB-expressing colon cancer cells and facilitating pyroptosis. Additionally, it’s crucial to examine the effects of pyroptosis on the diverse immune cells, inflammatory mediators, and signaling cascades within the TME of colon cancer. Future endeavors should also emphasize the integration of GSDMB-targeted therapies with chemotherapy or other immunotherapeutic modalities, striving to augment treatment efficacy.

## Data Availability

No datasets were generated or analysed during the current study.

## Abbreviations

CK: Cytokeratin
CRC: Colorectal cancer
CRS: Cytokine release syndrome
CTLA-4: Cytotoxic T lymphocyte-associated antigen-4
DAPI: 4’,6-diamidino-2-phenylindole
DCs: Dendritic cells
DEGs: Differentially expressed genes
dMMR: Mismatch-repair deficiency
GEO: Gene Expression Omnibus
GSDMB: Gasdermin B
GSEA: Gene Set Enrichment Analysis
GZMA: Granzyme A
GZMs: Granzymes
HRP: Horseradish peroxidase
ICIs: Immune checkpoint inhibitors
IFN-γ: Interferon-γ
MDSCs: Myeloid-derived suppressor cells
MHC-II: Major histocompatibility complex class II
mIHC: Multi-color immunohistochemistry
MSI-H: High microsatellite instability
MSI-L: Low microsatellite instability
MSS: Microsatellites stable
NK cells: Natural killer cells
OS: Overall survival
PCA: Principal component analysis
PCs: Principal components
PD-1: Programmed cell death-1
pMMR: Proficient mismatch-repair
scRNA-seq: Single-cell RNA sequencing
SNN: Shared nearest neighbor
ST: Spatial transcriptome
TCR: T-cell receptors
TGF-β: Transforming growth factor-β
TILs: Tumor-infiltrating lymphocytes
TMA: Tissue microarray
TME: Tumor microenvironment
TNF-α: Tumor necrosis factor-α
Tregs: Regulatory T cells
UMAP: Uniform manifold approximation and projection
VEGFA: Vascular endothelial growth factor A

## References

[1] Al Zein M, Boukhdoud M, Shammaa H, Mouslem H, El Ayoubi LM, Iratni R, et al. Immunotherapy and immunoevasion of colorectal cancer. Drug Discov Today. 2023;28(9):103669. 37328052 10.1016/j.drudis.2023.103669

[2] Shin AE, Giancotti FG, Rustgi AK. Metastatic colorectal cancer: mechanisms and emerging therapeutics. Trends Pharmacol Sci. 2023;44(4):222–36. 36828759 10.1016/j.tips.2023.01.003 PMC10365888

[3] Lin KX, Istl AC, Quan D, Skaro A, Tang E, Zheng X. PD-1 and PD-L1 inhibitors in cold colorectal cancer: challenges and strategies. Cancer Immunol Immunother. 2023;72(12):3875–93. 37831146 10.1007/s00262-023-03520-5 PMC10700246

[4] Ganesh K, Stadler ZK, Cercek A, Mendelsohn RB, Shia J, Segal NH, et al. Immunotherapy in colorectal cancer: rationale, challenges and potential. Nat Rev Gastroenterol Hepatol. 2019;16(6):361–75. 30886395 10.1038/s41575-019-0126-x PMC7295073

[5] Lichtenstern CR, Ngu RK, Shalapour S, Karin M. Immunotherapy, inflammation and colorectal cancer. Cells. 2020;9(3):618. 32143413 10.3390/cells9030618 PMC7140520

[6] Feliu V, Gomez-Roca C, Michelas M, Thébault N, Lauzéral-Vizcaino F, Salvioni A, et al. Distant antimetastatic effect of enterotropic colon cancer-derived α4β7^+^CD8^+^ T cells. Sci Immunol. 2023;8(84):eadg8841. 37289857 10.1126/sciimmunol.adg8841

[7] Joseph R, Soundararajan R, Vasaikar S, Yang F, Allton KL, Tian L, et al. CD8^+^ T cells inhibit metastasis and CXCL4 regulates its function. Br J Cancer. 2021;125(2):176–89. 33795809 10.1038/s41416-021-01338-5 PMC8292398

[8] Ding L, Sun L, Bu MT, Zhang Y, Scott LN, Prins RM, et al. Antigen presentation by clonally diverse CXCR5 + B cells to CD4 and CD8 T cells is associated with durable response to immune checkpoint inhibitors. Front Immunol. 2023;14:1176994. 37435085 10.3389/fimmu.2023.1176994 PMC10330698

[9] Ouyang X, Zhou J, Lin L, Zhang Z, Luo S, Hu D. Pyroptosis, inflammasome, and gasdermins in tumor immunity. Innate Immun. 2023;29(1–2):3–13. 36632024 10.1177/17534259221143216 PMC10164276

[10] Voskoboinik I, Whisstock JC, Trapani JA. Perforin and granzymes: function, dysfunction and human pathology. Nat Rev Immunol. 2015;15(6):388–400. 25998963 10.1038/nri3839

[11] Li L, Jiang M, Qi L, Wu Y, Song D, Gan J, et al. Pyroptosis, a new bridge to tumor immunity. Cancer Sci. 2021;112(10):3979–94. 34252266 10.1111/cas.15059 PMC8486185

[12] Zhou Z, He H, Wang K, Shi X, Wang Y, Su Y, et al. Granzyme A from cytotoxic lymphocytes cleaves GSDMB to trigger pyroptosis in target cells. Science. 2020;368(6494):eaaz7548. 32299851 10.1126/science.aaz7548

[13] Oltra SS, Colomo S, Sin L, Pérez-López M, Lázaro S, Molina-Crespo A, et al. Distinct GSDMB protein isoforms and protease cleavage processes differentially control pyroptotic cell death and mitochondrial damage in cancer cells. Cell Death Differ. 2023;30(5):1366–81. 36899106 10.1038/s41418-023-01143-y PMC10154425

[14] Du T, Gao J, Li P, Wang Y, Qi Q, Liu X, et al. Pyroptosis, metabolism, and tumor immune microenvironment. Clin Transl Med. 2021;11(8):e492. 34459122 10.1002/ctm2.492 PMC8329701

[15] Jia Y, Wang X, Deng Y, Li S, Xu X, Qin Y, et al. Pyroptosis provides new strategies for the treatment of cancer. J Cancer. 2023;14(1):140–51. 36605484 10.7150/jca.77965 PMC9809330

[16] Jorgovanovic D, Song M, Wang L, Zhang Y. Roles of IFN-gamma in tumor progression and regression: a review. Biomark Res. 2020;8:49. 33005420 10.1186/s40364-020-00228-x PMC7526126

[17] Li L, Li Y, Bai Y. Role of GSDMB in pyroptosis and cancer. Cancer Manag Res. 2020;12:3033–43. 32431546 10.2147/CMAR.S246948 PMC7201009

[18] Ivanov AI, Rana N, Privitera G, Pizarro TT. The enigmatic roles of epithelial gasdermin B: recent discoveries and controversies. Trends Cell Biol. 2023;33(1):48–59. 35821185 10.1016/j.tcb.2022.06.006 PMC9789163

[19] Gámez-Chiachio M, Molina-Crespo Á, Ramos-Nebot C, Martinez-Val J, Martinez L, Gassner K, et al. Gasdermin B over-expression modulates HER2-targeted therapy resistance by inducing protective autophagy through Rab7 activation. J Exp Clin Cancer Res. 2022;41(1):285. 36163066 10.1186/s13046-022-02497-w PMC9511784

[20] Pelka K, Hofree M, Chen JH, Sarkizova S, Pirl JD, Jorgji V, et al. Spatially organized multicellular immune hubs in human colorectal cancer. Cell. 2021;184(18):4734–52. 34450029 10.1016/j.cell.2021.08.003 PMC8772395

[21] Valdeolivas A, Amberg B, Giroud N, Richardson M, Gálvez EJC, Badillo S et al. Charting the heterogeneity of colorectal cancer consensus molecular subtypes using spatial transcriptomics. bioRxiv. 2023:2023.2001.2023.525135. 10.1038/s41698-023-00488-4 PMC10781769 38200223

[22] Tan Y, Chen Q, Li X, Zeng Z, Xiong W, Li G, et al. Pyroptosis: a new paradigm of cell death for fighting against cancer. J Exp Clin Cancer Res. 2021;40(1):153. 33941231 10.1186/s13046-021-01959-x PMC8091792

[23] Vinay DS, Ryan EP, Pawelec G, Talib WH, Stagg J, Elkord E, et al. Immune evasion in cancer: mechanistic basis and therapeutic strategies. Semin Cancer Biol. 2015;35(Suppl):S185–198. 25818339 10.1016/j.semcancer.2015.03.004

[24] Huang C, Ren S, Chen Y, Liu A, Wu Q, Jiang T, et al. PD-L1 methylation restricts PD-L1/PD-1 interactions to control cancer immune surveillance. Sci Adv. 2023;9(21):eade4186. 37235656 10.1126/sciadv.ade4186 PMC10219601

[25] Tian LY, Smit DJ, Jücker M. The role of PI3K/AKT/mTOR signaling in hepatocellular carcinoma metabolism. Int J Mol Sci. 2023;24(3):2652. 36768977 10.3390/ijms24032652 PMC9916527

[26] Shiau JP, Chuang YT, Cheng YB, Tang JY, Hou MF, Yen CY, et al. Impacts of oxidative stress and PI3K/AKT/mTOR on metabolism and the future direction of investigating fucoidan-modulated metabolism. Antioxid (Basel). 2022;11(5):911. 10.3390/antiox11050911 PMC9137824 35624775

[27] Efremova M, Vento-Tormo M, Teichmann SA, Vento-Tormo R. CellPhoneDB: inferring cell-cell communication from combined expression of multi-subunit ligand-receptor complexes. Nat Protoc. 2020;15(4):1484–506. 32103204 10.1038/s41596-020-0292-x

[28] Peng C, Huggins MA, Wanhainen KM, Knutson TP, Lu H, Georgiev H, et al. Engagement of the costimulatory molecule ICOS in tissues promotes establishment of CD8^+^ tissue-resident memory T cells. Immunity. 2022;55(1):98–114. 34932944 10.1016/j.immuni.2021.11.017 PMC8755622

[29] Liu Z, Zhou Q, Wang Z, Zhang H, Zeng H, Huang Q, et al. Intratumoral TIGIT^+^ CD8^+^ T-cell infiltration determines poor prognosis and immune evasion in patients with muscle-invasive bladder cancer. J Immunother Cancer. 2020;8(2):e000978. 32817209 10.1136/jitc-2020-000978 PMC7430558

[30] Freed-Pastor WA, Lambert LJ, Ely ZA, Pattada NB, Bhutkar A, Eng G, et al. The CD155/TIGIT axis promotes and maintains immune evasion in neoantigen-expressing pancreatic cancer. Cancer Cell. 2021;39(10):1342–60. 34358448 10.1016/j.ccell.2021.07.007 PMC8511341

[31] Dixon KO, Tabaka M, Schramm MA, Xiao S, Tang R, Dionne D, et al. TIM-3 restrains anti-tumour immunity by regulating inflammasome activation. Nature. 2021;595(7865):101–6. 34108686 10.1038/s41586-021-03626-9 PMC8627694

[32] Juneja VR, McGuire KA, Manguso RT, LaFleur MW, Collins N, Haining WN, et al. PD-L1 on tumor cells is sufficient for immune evasion in immunogenic tumors and inhibits CD8 T cell cytotoxicity. J Exp Med. 2017;214(4):895–904. 28302645 10.1084/jem.20160801 PMC5379970

[33] Ma J, Yan S, Zhao Y, Yan H, Zhang Q, Li X. Blockade of PD-1 and LAG-3 expression on CD8^+^ T cells promotes the tumoricidal effects of CD8^+^ T cells. Front Immunol. 2023;14:1265255. 37841254 10.3389/fimmu.2023.1265255 PMC10568325

[34] Banta KL, Xu X, Chitre AS, Au-Yeung A, Takahashi C, O’Gorman WE, et al. Mechanistic convergence of the TIGIT and PD-1 inhibitory pathways necessitates co-blockade to optimize anti-tumor CD8^+^ T cell responses. Immunity. 2022;55(3):512–26. 35263569 10.1016/j.immuni.2022.02.005 PMC9287124

[35] Farhood B, Najafi M, Mortezaee K. CD8^+^ cytotoxic T lymphocytes in cancer immunotherapy: a review. J Cell Physiol. 2019;234(6):8509–21. 30520029 10.1002/jcp.27782

[36] Jia X, Chua BY, Loh L, Koutsakos M, Kedzierski L, Olshansky M, et al. High expression of CD38 and MHC class II on CD8^+^ T cells during severe influenza disease reflects bystander activation and trogocytosis. Clin Transl Immunol. 2021;10(9):e1336. 10.1002/cti2.1336 PMC8426257 34522380

